# Sequence analysis for detection of drug resistance in *Mycobacterium tuberculosis* complex isolates from the Central Region of Cameroon

**DOI:** 10.1186/1471-2180-14-113

**Published:** 2014-05-03

**Authors:** Emmanuel Mouafo Tekwu, Larissa Kamgue Sidze, Jean-Paul Assam Assam, Jean-Claude Tedom, Serges Tchatchouang, Gaëlle Guiewi Makafe, Anne-Laure Tchokote Wetewale, Christopher Kuaban, Sara Eyangoh, Francine Ntoumi, Véronique N Penlap Beng, Matthias Frank

**Affiliations:** 1 Laboratory for Tuberculosis Research (LTR), Biotechnology Centre (BTC)-Nkolbison, University of Yaoundé I, Yaoundé, Cameroon; 2 Pneumology unit, Jamot Hospital, Yaoundé, Cameroon; 3 Mycobacteriology Service, Reference laboratory of NTP, Centre Pasteur of Cameroon-Pasteur Institute International Network, Yaoundé, Cameroon; 4 Fondation Congolaise pour la Recherche Médicale, Université Marien Gouabi, Brazzaville, Congo; 5 Institute for Tropical Medicine, University of Tübingen, Tübingen, Germany; 6 Central African Network for Tuberculosis, AIDS/HIV and Malaria (CANTAM), Brazzaville, Congo; 7 Deutsches Zentrum für Infektionsforschung (DZIF), Tübingen, Germany

**Keywords:** Phenotype, Mutation, Drug resistance, *Mycobacterium tuberculosis*, Cameroon

## Abstract

**Background:**

The potential of genetic testing to rapidly diagnose drug resistance has lead to the development of new diagnostic assays. However, prior to implementation in a given setting, the association of specific mutations with specific drug resistance phenotypes should be evaluated. The purpose of this study was to evaluate molecular markers in predicting drug resistance in the Central Region of Cameroon.

**Results:**

From April 2010 and March 2011, 725 smear positive pulmonary tuberculosis patients were enrolled and all positive cultures were tested for drug susceptibility. A total of 63 drug resistant and 100 drug sensitive *Mycobacterium tuberculosis* complex clinical isolates were screened for genetic mutations in *katG*, *inhA*, *ahpC*, *rpoB*, *rpsL*, *rrs, gidB* and *embCAB* loci using DNA sequencing. Of the 44 isoniazid resistant (INH^R^) isolates (24 high level, 1 μg/ml and 20 low level, 0.2 μg/ml), 73% (32/44) carried the *katG315* and/or the -*15 inhA* promoter mutations. Of the 24 high level INH^R^, 17 (70.8%) harbored *katG315* mutation, 1 a point mutation (-15C → T) in the *inhA* promoter and 6 were (25.0%) wild types. Thus, for INH^R^ high level detection, *katG315* mutation had a specificity and a sensitivity of 100% and 70.8% respectively. Of the 20 low level INH^R^, 10 (50.0%) had a -15C → T mutation in the *inhA* promoter region, and 1 (2.2%) a -32G → A mutation in the *ahpC* promoter region. All of the 7 rifampicin resistant (RIF^R^) isolates carried mutations in the *rpoB* gene (at codons Ser531Leu (71.4%), His526Asp (14.3%), and Asp516Val (14.3%)). Of the 27 streptomycin resistant (SM^R^) isolates, 7 carried mutations at the *rpsL* and the *gidB* genes. 1 of the 2 ethambutol resistant *(EMB*^R^) isolates displayed a mutation in *embB* gene.

**Conclusion:**

This study provided the first molecular investigation assessing the correlation of phenotypic to genotypic characteristics on MTB isolates from the Central Region of Cameroon using DNA sequencing. Mutations on *rpoB*, *katG315* and -15 point mutations in *inhA* promoter loci could be used as markers for RIF and INH -resistance detection respectively.

## Background

Despite the availability of an effective treatment for decades, tuberculosis (TB) continues to cause great mortality and suffering, especially in poor and less-developed countries. Its association with the HIV/AIDS pandemic forms a lethal combination. In addition, multidrug resistant (MDR) TB and the recently-described extensively drug resistant (XDR) TB severely complicate the management and control of the disease worldwide [1,2]. Almost 8.8 million new cases of TB were reported in 2010, and 1.4 million deaths were attributed to the disease. Asia and Sub-Saharan Africa accounted for 85% of new cases of TB worldwide [3]. Of the 8.8 million incident cases in 2010, 1.1 million (13%) were among people living with HIV.

Tuberculosis remains a common disease in Cameroon, with an estimated of 25 000 cases annually [4]. Like in other poor resources countries, therapeutic decisions are most often made by algorithms according to WHO guidelines. Inadequate or improper prescription of drugs, poor patient compliance and supervision of therapy may lead to the emergence of drug resistant strains [5]. Drug resistance in tuberculosis (TB) is a matter of great concern for TB control programs since these strains could spread in the community, stressing the need for early detection of drug resistance and subsequently initiation of adjusted therapy. Conventional diagnosis of drug-resistance in MTB strains relies heavily upon mycobacterial culture and drug susceptibility testing in liquid or solid media. Usually, results are only obtained after weeks to months of incubation and many developing countries lack the resources to establish the stringent laboratory conditions needed for these growth-based methods. From a clinical perspective, the existing growth-based diagnostics are too slow as patients undergoing treatment with drugs to which they are resistant, remain contagious, and those with XDR-TB and HIV often die before they are even diagnosed [6]. Major advances in molecular biology and the availability of new information generated after deciphering the complete genome sequence of *M. tuberculosis*[7], have led to the development of new tools for rapid detection of drug resistance [8,9]. Molecular methods are based on assigning the presence or absence of certain mutations in specific positions or genetic locations which are known to be associated with resistance [10]. About 95% of rifampicin (RIF) -resistant strains have mutations in the 81-bp core region of the *rpoB* gene encoding the β-subunit of the RNA polymerase, named *RIF-Resistance Determining Region* (RRDR) [11]. In contrast to RIF, the situation for isoniazid (INH) is much more complex. Resistance mutations have been reported in at least 4 different genes including *katG, inhA, ahpC* and *oxyR*[10]. Meanwhile, resistance against streptomycin (SM) has been reported to be associated with mutations in *rrs* gene, which codes for 16S ribosomal RNA, and *rpsL* coding for the ribosomal protein S12 [12] and these mutations are found in a limited proportion of clinically isolated SM-resistant *M. tuberculosis* strains. Recently, Okamoto et al. [13] found that mutations within the *gidB* gene which encodes a conserved 7-methylguanosine (m^7^G) methyltransferase specific for the 16S rRNA, is associated with low-level SM-resistance and are an important cause of resistance found in 33% of resistant *M. tuberculosis* isolates. Resistance to ethambutol (EMB) is primarily mediated by mutations in the *embB* gene, coding for an arabinosyltransferase participating in mycobacterial cell wall synthesis, with codon 306 being most frequently affected [14]. Furthermore, mutations in the *embA*[15,16] and upstream of *embC*[16,17] are also involved in EMB -resistance.

Since the frequency and type of gene mutation varies greatly among different geographic regions in the world [18], it is important to evaluate the type and distribution of resistance associated mutations as a prerequisite for a large-scale implementation of genotypic approaches aimed at rapidly detecting resistance. However, up to now data assessing sensitivity and specificity of specific mutations for the detection of drug resistance phenotypes in our settings is still unavailable. Therefore CANTAM (Central Africa Network for Tuberculosis, HIV/AIDS and Malaria) an EDCTP (European and Developing Clinical Trials Partnership) funded network [19], with the goal to establish a cohort and prepare new sites for conducting future clinical trials of new TB drugs and vaccines in Central Africa countries, carried out a population based study, involving MTBC strains from Central region of Cameroon, to determine the genetic basis of first line drug resistance.

## Methods

### Mycobacterial isolates

During this baseline study carried out between April 2010 and March 2011, 725 smear positive pulmonary tuberculosis patients were enrolled at Jamot Hospital and Mbalmayo District Hospital. All positive cultures were tested for drug susceptibility to INH (0.2 μg/ml and 1 μg/ml), RIF (40 μg/ml), EMB (2 μg/ml), SM (4 μg/ml), OFX (2 μg/ml) and KAN (20 μg/ml) by the indirect proportion method on Lowenstein Jensen medium [20]. Phenotypically, 44 isolates were INH^R^ (24 high level and 20 low level), 27 isolates were SM^R^, 7 isolates were RIF^R^ and 2 isolates were EMB^R^. The 63 resistant isolates to INH, RIF, SM and EMB or MDR were screened for genetic mutations. In addition, *M. tuberculosis* strain H37Rv (susceptible) and 100 fully susceptible clinical isolates from the panel of susceptible strains collected during the study period were included to serve as controls. The study was approved by the Cameroon National Ethic Committee and the Cameroonian Ministry of Public Health. Written informed consent was obtained from all study subjects.

### DNA extraction

Briefly, a loop-full of mycobacterial colonies was suspended in 400 μl of 10 mM Tris–HCl, 1 mM EDTA (pH 7.0) buffer and inactivated at 90°C for 30 min. The suspension was then centrifuged at 12,000 g for 1 min and the supernatant, containing nucleic acids, was harvested and transferred into a new eppendorf tube. Crude DNA extracts were stored at -20°C and then shipped to Germany for molecular analysis according to International Air Transport Association guidelines.

### PCR amplification of target genes

The DNA extract was used as a template for PCR with the primers listed in Table 1. Each final PCR volume of 20 μl contained 10× PCR buffer (Qiagen, Germany), 5% DMSO, 20 pmol of forward and 20 pmol of reverse primers, 11.9 μl of distilled water, 0.5 μl MgCl_2_ 25 mM (Qiagen, Germany), dNTPs at a final concentration of 500 μM, 0.2 μl of *Taq polymerase* 5 U/μL (Qiagen, Germany), and 2 μl of crude DNA extract (≈50 ng). The cycling program included a cycle of an initial denaturation step at 94°C for 5 min, followed by 35 cycles of denaturation at 94°C for 1 min, annealing at the temperature and time indicated in Table 1, and elongation at 72°C for 1 min. The final elongation step was set at 72°C for 10 min for one cycle. The PCR products were examined by gel electrophoresis and purified by use of a Nucleospin Extract II kit (Macherey Nagel, Germany) according to the manufacturer instructions.

**Table 1:** Primers used in the study

**Loci**	**Primers**	**Sequences**	**Annealing T° (time)**	**Expected size**	**Reference**
*katG*	F	5′-GAAACAGCGGCGCTGATCGT-3′	66°C (1 min)	210 bp	[21]
	R	5′- GTTGTCCCATTTCGTCGGGG- 3′			
*fabGI-inhA*	F	5′-CCTCGCTGCCCAGAAAGGGA-3′	64°C (1 min)	248 bp	[21]
	R	5′-ATCCCCCGGTTTCCTCCGGT-3′			
*inhA (ORF)*	F	5′- GAACTCGACGTGCAAAAC – 3′	55°C (45 sec)	207 pb	[18]
	R	5′- CATCGAAGCATACGAATA – 3′			
*ahpC*	F	5′-ACCACTGCTTTGCCGCCACC-3′	65°C (1 min)	237 bp	[21]
	R	5′-CCGATGAGAGCGGTGAGCTG-3′			
*rpoB*	F	5′-TCGCCGCGATCAAGGAGT-3′	62°C (30 sec)	158 bp	[21]
	R	5′-GTGCACGTCGCGGACCTCCA-3′			
*rrs530*	F	5′-GATGACGGCCTTCGGGTTGT-3′	62°C (1 min)	238 bp	[12]
	R	5′- TCTAGTCTGCCCGTATCGCC -3′			
*rrs912*	F	5′- GTAGTCCACGCCGTAAACGG -3′	62°C (1 min)	240 bp	[12]
	R	5′- AGGCCACAAGGGAACGCCTA -3′			
*rpsL*	F	5′- GGCCGACAAACAGAACGT -3′	58°C (30 sec)	375 bp	[12]
	R	5′- GTTCACCAACTGGGTGAC -3′			
*embC*	F	5′- GTTCGACAAGCGCGCCACAC -3′	65°C (45 sec)	334 bp	[22]
	R	5′- CGGAGGTAGATGGTAGCCGG -3′			
*embA*	F	5′- GCCGGCTATGTAGCCAACTA -3′	65°C (45 sec)	338 bp	[17]
	R	5′- GACCGTTCCACCAACACC -3′			
*embB*	F	5′- CCGACCACGCTGAAACTG -3′	65°C (45 sec)	368 bp	[23]
	R	5′- GTAATACCAGCCGAAGGGATCCT -3′			
*gidB*	F	5′-CGCCGAGTCGTTGTGCT-3′	62°C (1 min)	886 pb	-
	R	5′-AGCCTGGCCCGACCTTA-3′			

T° = Temperature.

### Sequencing

Purified PCR products were sequenced with the same primers using the ABI’s Big dye terminator kit (Applied Biosystems, USA) according to the manufacturer’s instructions. At each locus, both forward and reverse primers at each locus were included in order to maximize the coverage of the amplified gene fragment, and the reproducibility of the results. Sequencing reactions include 1 μl big dye, 2 μl sequencing buffer, 0.5 μl of each 2.5 μM primer, a volume of PCR template corresponding approximately to 2–3 ng of DNA, and sufficient distilled water for obtaining a 10 μl final volume. Unincorporated terminators were removed by treatment on a sephadex column. The obtained sequences were aligned using the assembling application of vector NTI (Invitrogen) and *CodonCode Aligner*, and polymorphisms detection was achieved by comparison with the published *M. tuberculosis* H37Rv sequence.

### Quality control

*M. tuberculosis* H37Rv (ATCC 27294) was included as a quality controls for the phenotypic and genotypic tests.

## Results

### Analysis of INH -resistance associated mutation

A total of 44 INH^R^ (24 high level and 20 low level)) and 100 matched INH^S^ sensitive control strains were screened for mutations at *katG* codon 315, the *fabG1-inhA* regulatory region, the *inhA* ORF, the *oxyR-ahpC* intergenic region by DNA sequence analysis. A complete list of specific mutations, which had been identified is provided in Table 2.

**Table 2:** **Isoniazid resistance- associated mutations detected in *M. tuberculosis *** **study isolates**

**Gene**	**N° of isolates tested**	**N° of isolates with indicated genotype**	**Nucleotide change**	**Amino acid change**	**N° of mutated isolates according to INH resistance level**	
					Low level (0.2 μg/ml)	High level (1 μg/ml)
*katG*	44 INH^R^	18	315AGC → ACC	Ser → Thr	2	16
		1	315AGC → AAC	Ser → Asn	0	1
		1	Partial deletion	NA	0	1
		24	WT	NA	0	0
	100 INH^S^	0	WT	NA	NA	NA
*fabG1-inhA regulatory region*	44 INH^R^	13	-15C → T	NA	10	3
		5	-47G → C	NA	0	5
		26	WT	NA	0	0
	100 INH^S^	24	-47G → C	NA	NA	NA
		3	-102C → T	NA	NA	NA

NA = not applicable; WT = wild type; INH^R^ = isoniazid resistant isolate; INH^S^ = isoniazid sensitive isolate; N°: number.

### Polymorphisms in the *katG* gene

Among the 24 high level INH-resistant isolates, 18 (75%) were genetically altered in the *katG* region. Out of these, 17 (70.8%) had a resistance associated mutation in *katG* codon 315 and one isolate had a partial *katG* gene deletion (Table 2).

Of the 18 isolates altered in the *katG* gene, 7 had an additional mutation in the *fabG1-inhA* regulatory region (2 at position -15C → T and 5 at position -47G → C). The *katG315* mutations resulted in a change of the wild-type codon, AGC (Ser) to ACC (Thr) in 17 strains and AAC (Asn) in one strain. All of the INH susceptible strains lacked mutations in *katG 315*. Thus for detection of high level INH -resistance, mutation/partial deletion of the *katG* gene had a specificity of 100.0% and a sensitivity of 75% (18/44).

Of the 20 low level INH-resistant isolates, 2 (10%) harboured the *katG315* mutation. In total, the *katG315* mutation was seen in 19 isolates with 16 (84.2%) being high level INH-resistant isolates. Therefore, this mutation might be associated with high level INH -resistance (1 μg/ml). Overall, for the detection of INH -resistance, mutation/partial deletion of the *katG* gene had a specificity of 100.0% and a sensitivity of 45.5% (20/44).

### Polymorphisms in the *inhA* gene

The *inhA* region consists of two genes, *fabG1* and *inhA.* Among the 24 high level INH-resistant isolates, 3 harboured the mutation -15C → T in the regulatory region of *inhA* with 2 of them carrying an additional *katG315* mutation and 5 had nucleotide changes (G → C) at position -47. All the 5 INH-resistant isolates with -47 G → C mutation also harbored the *katG*315 mutation.

Out of the 20 low level INH-resistant isolates, 10 (50%) had mutations in *fabG1-inhA* leading to a C → T change at position -15 of the start site of *fabG*. In total, the *fabG1-inhA* mutation at -15 position was observed in 13 isolates with 10 (77%) being low level INH-resistant isolates. Therefore, this mutation seems to be associated with low level INH -resistance (0.2 μg/ml). None of the INH susceptible isolates had the mutation affecting the *inhA* promoter region at position -15. On the contrary, the nucleotide change at position -47 was also seen in 24 isoniazid susceptible isolates and a new mutation -102C → T not yet described was detected in 3 other INH susceptible isolates. No mutation was observed in *inhA ORF* gene (Table 3).

**Table 3:** **Rifampicin resistance-associated mutations detected in *M. tuberculosis *** **study isolates**

**Gene**	**N° and type of isolates tested**	**N° of isolates with indicated genotype**	**Nucleotide change**	**Amino acid change**
*rpoB*	7 RIF^R^	5	531TCG → TTG	Ser → Leu
		1	526CAC → GAC	His → Asp
		1	516GAC → GTC	Asp → Val
	100 RIF^S^	1	516GAC → TAC	Asp → Tyr

NA = not applicable; WT = wild type; RIF^R^ = rifampicin resistant isolate; RIF^S^ = rifampicin sensitive isolate, N° = Number.

### Polymorphisms in the *oxyR-ahpC* intergenic region

One low level INH-resistant isolate displayed a G → A substitution at position 32 upstream of the transcriptional start site of *ahpC* in the *oxyR-ahpC* intergenic region, which has previously been shown to be involved in INH -resistance [15].

### Combined sensitivity and specificity of *katG* and *inhA* promoter region for INH resistance

Mutations in *katG315* and -*15*C → T in *inhA* promoter region accounted together for 73% (33/44) INH -resistance. Since none of these mutations was observed in susceptible isolates, the combined specificity is 100%.

### Analysis of the *rpoB* gene responsible for RIF-resistance

In this study, 7 RIF^R^ isolates, and 100 RIF-sensitive (RIF^s^) clinical isolates were examined for mutations in a 158-bp fragment of *rpoB* gene. Of 7 RIF^R^ isolates, resistance-associated mutations in the core region of *rpoB* were found in all 7 (100.0%) isolates (Table 3). The nucleotide and amino acid changes identified in drug-resistant isolates are shown in Table 4. Three different *rpoB* mutations were identified involving codons 516, 526, and 531. The most common mutation, which changes TCG (Ser) to TTG (Leu) in codon 531, was detected in 5 (71.4%) of the 7 mutated RIF-resistant isolates (Table 3). A mutation affecting codon 516 and leading to a substitution of aspartate to tyrosine was observed in the *rpoB* gene of one RIF sensitive isolate. Hence, mutations in the *rpoB* gene exhibited a sensitivity of 100.0% and a specificity of 99.0%.

**Table 4:** **Streptomycin and ethambutol resistance-associated mutations detected in *M. tuberculosis *** **study isolates**

**Resistance to**	**Gene**	**N° and type of isolates tested**	**N° of isolates with indicated genotype**	**Nucleotide change**	**Amino acid change**
**Streptomycin**	*rpsL*	27 SM^R^	2	43AAG → AGG	Lys → Arg
		100 SM^S^	0	WT	NA
	*gidB*	27 SM^R^	1	138GCG → CCG	Ala → Pro
			1	79TTG → TGG	Leu → Trp
			1	75CCG → TCG	Pro → Ser
			1	48CAT → AAT	His → Asn
			1	36GTG → GGG	Val → Gly
		100 SM^S^	3	205GCA → GCG	Ala → Ala*
			3	16CTT → CGT	Leu → Arg
**Ethambutol**	*embC*	2 EMB^R^	0	WT	NA
		100 EMB^S^	3	-20A → C	NA
			3	-230A → C	NA
	*embA*	2 EMB^R^	0	WT	NA
		100 EMB^S^	3	330CTG → TTG	Leu → Leu*
	*embB*	2 EMB^R^ 100 EMB^S^	1	306	Met → Val
			0	WT	NA

*: synonymous mutation; NA = not applicable; WT = wild type; SM^R^ = streptomycin resistant isolate; SM^S^ = streptomycin sensitive isolate; EMB^R^ = ethambutol resistant isolate; EMB^S^ = ethambutol sensitive isolate; N° = Number.

### Analysis of mutations in the target regions of SM -resistance

All strains were first sequenced (27 SM^R^ isolates and 100 fully susceptible isolates) in the *rrs* gene. As none of the resistant strains displayed a mutation in this gene, sequence analysis of *rpsL* was performed. Among the 27 SM^R^ strains 2 carried a mutation in *rpsL* gene at codon 43 and none showed a polymorphism at codon 88. The 2 resistant isolates mutated at codon 43 had a Lys → Arg substitution (Table 4). The remainder of the phenotypically resistant strains (n = 25) did not carry a mutation in *rpsL* gene and no changes were found in the drug-susceptible isolates. The specificity of *rpsL43* mutation for resistance detection of SM^R^ was 100%.

Additionally all strains were sequenced in *gidB* gene. In this very polymorphic gene, 5 different mutations with 3 of them never been reported were found in 5 SM^R^ strains and 2 different mutations in 6 SM^S^ strains (see Table 4). The 5 mutations at codon 36GTG → GGG, 48CAT → AAT, 75CCG → TCG, 79TTG → TGG, 138GCG → CCG were exclusively found in streptomycin resistant strains while the mutations at codon 205GCA → GCG and 16CTT → CGT were exclusively found in streptomycin sensitive strains.

### Analysis of mutations in the target regions of EMB -resistance

In this study, we analyzed polymorphisms in the *embCAB* operon for 2 ethambutol resistant isolates and 100 ethambutol sensitive isolates. Among our 2 EMB -resistant isolates, sequence analysis of the *embB* gene identified 1 isolate with EMB-resistance-associated nucleotide substitutions in codon 306ATG → GTG that result in amino acid replacement (306Met → Val) and the remaining isolate as well as all sensitive isolates had no amino acid replacements in *embB* gene. As *embB* mutations are not the only ones involved in EMB-resistance mechanisms in *M. tuberculosis*, we also analyzed *embC* and *embA* loci for mutations. Sequence analyses of *embC* and *embA* revealed no mutations in EMB -resistant isolates while 6 of 100 fully susceptible isolates have mutations at position -20A → C and -230A → C of *embC* upstream region. Although the substitutions at position -20A → C were present only in EMB-susceptible organisms in our sample, these three strains also had synonymous mutation at codon 330CTG → TTG of the *embA* gene and nucleotides replacement at position -102C → T in the regulatory region of *fabG1-inhA* operon; this is exclusively found in susceptible organisms. The 3 samples with mutations at position -230A → C also harbored simultaneously a nucleotide replacement at position -47 in the regulatory region of *fabG1-inhA* operon. Three of 100 fully susceptible strains had synonymous mutations at codon 330CTG → TTG of *embA* gene, which did not resulting in amino acid replacement. These 3 isolates harbored simultaneously nucleotide replacement at position -20 upstream of the initiation site of *embC* gene. All EMB susceptible strains (n = 100) had a wild-type *embB* sequence.

## Discussion

Early detection of drug resistance constitutes one of the priorities of TB control programs. It allows initiation of the appropriate treatment in patients and avoids dissemination of resistant strains in the community. In the context of a poor resource country, detection of drug resistance is performed in the reference laboratory by so-called ‘conventional methods’ based on detection of growth of *M. tuberculosis* in the presence of the respective antibiotics. Depending on the method, this process requires at least 10 days to 8 weeks before drug sensitivity results are available. During this time the infected patient may be treated incorrectly which may have serious health implications in particular in patients with HIV-TB coinfection. The disclosure of the genetic basis of resistance to anti-tuberculous agents has enabled development of new molecular tests to detect mutations associated with reduced susceptibility to antituberculous drugs [9,10]. In order to detect and validate the drug resistance associated mutations, DNA sequencing is the most accurate among the molecular techniques. We used PCR fragment sequencing since molecular mechanisms explaining resistance to anti-tuberculous agents are not fully understood [24]. It presents the advantage, over methods that use DNA probes, to detect unknown mutations. Recently the GeneXpert has been endorsed by the WHO for point of care testing [25]. Drug sensitivity testing with this method is based on the detection of mutations in the core region of the *rpoB* gene, thus only RIF-resistance or MDR would be detected.

In this study, we set out to investigate the association of phenotypic resistance with genetic mutations in drug resistance TB isolates in Cameroon. The majority of the isolates in this study were from the Jamot Hospital (Central Region of Cameroon), the reference hospital for diagnostic and treatment of pulmonary diseases throughout the country. Therefore, the data obtained in this study can be considered to be representative of the make-up of resistance conferring mutations present in *M. tuberculosis* strains in this region.

A 158-bp fragment of the *rpoB* gene from codon 507 to 533 was amplified and sequenced to detect mutations in RIF^R^ strains. Of the 7 phentotypically RIF^R^ strains, mutations were found in the rifampicin resistant determining region (*RRDR)* for all the 7 isolates. These alterations affected the codons Ser531Thr (71.4%), His526Asp (14.3%) and Asp516Val (14.3%). The *rpoB* codons 531, 526, and 516 are the most frequently mutated codons worldwide, although variations in the relative frequencies of mutations in these codons have been described for *M. tuberculosis* isolates from different geographic locations. The most common site of nucleotide substitutions in RIF^R^ isolates was codon 531. This finding was similar to those reported in Russia [26], the US [27], Tunisia [28] Ghana [21] and Germany [29]. The codon 531 mutation was also reported as the most frequent (68%) in *M. tuberculosis* isolates of the LAM family in Cameroon [30]. For codons 526 and 516 involved in RIF^R^, mutations in our strains occurred at equal frequencies than in strains from other geographical regions [31-33]. It has been shown that various substitutions in the same codon can lead to different level of resistance. Mutations at codon 516 of the *rpoB* gene can confer either low or high level resistance depending on the codon change [34]. It has been reported that substitution of aspartate by tyrosine in codon 516 induced RIF-resistance of *M. tuberculosis* with minimum inhibitory concentration (MIC) between 15 μg/ml and 25 μg/ml in BACTEC 460-TB system [34]. In our study, RIF susceptibility was evaluated in Lowenstein Jensen at a concentration of 50 μg/ml. This might explain why strains harbouring this mutation in our study were phenotypically RIF-susceptible. Among the 7 isolates which were altered genetically, 6 were MDR strains and one a RIF-SM-resistant isolate. Thus, *rpoB* could be an indicator of multidrug resistance among *M. tuberculosis* strains. This observation was previously reported among Cameroonian *M. tuberculosis* isolates [30].

It has been previously shown that about 10–15.9% of RIF -resistant isolates do not have mutations in the RRDR [15]. More than 90% of RIF -resistant strains from other regions had mutations located in the 81-bp core region [35-38]. This indicated a possible occurrence of alteration outside the core region of 81 bp of the examined *rpoB*. Among other explanations, several additional genes might be involved in RIF-resistance such as *rpoA, rpoC* or *rpoD*[39]. The natural resistance to RIF in some *M. avium* and *M. intracellular* strains is known to be a result of efficient cell wall permeability and exclusion barrier, suggesting that these elements could also play an important role in *M. tuberculosis*[34]. However, in our study, all the isolates harboured mutations in the RRDR core region.

Common genes known to be involved in INH-resistance are *katG, inhA, ahpC, oxyR*[10]. Several investigators have shown that INH-resistance in *M. tuberculosis* isolates arise principally from a *katG* gene alteration [40-42] that corresponds essentially to point mutations in codon 315 (point mutations in two bases 944 and 945). In this study, 18 (40.0%) INH -resistant isolates were genetically altered in the *katG* codon 315. Others studies have reported 95% of all INH-resistant isolates with mutations in codon 315 [43]. Out of the 6 MDR strains identified in this study, 5 displayed a high level resistance to isoniazid with a *katG* alteration and the remaining one displayed a low level INH-resistance with -32G → A mutation in *oxyR-ahpC* intergenic region. Therefore, it will be useful to combine *katG315 and -15* point mutation *inhA* promoter region with *rpoB* in molecular assays looking at drug resistance. Since some of the INH^R^ strains in this study had no mutation in *katG*315 and -*15 inhA* promoter region, it is likely that mutations in other genes, such as the *inhA* locus, contribute to resistance. Previous studies have shown that mutations in the upstream region of the *inhA* locus result in increased levels of *InhA* (NADH-dependent enoyl-acyl carrier protein reductase) expression, thereby elevating the drug target levels and producing INH -resistance via a titration mechanism [15]. We assessed for mutations in the *inhA* regulatory region of all the 44 INH^R^*M. tuberculosis* strains and found a substitution at position 15 upstream of the start codon in 13 (28.9%) isolates. The frequency of the occurrence of specific INH-resistance conferring mutations varied between geographical regions in the world [26]. A study in Equatorial Guinea reported the absence of mutation in the *katG* gene of *M. tuberculosis* INH -resistant isolates [44]. The unique *katG* mutation observed in this study was the substitution of Serine to Threonine at codon 315. High proportion of Ser315*katG* mutations has been reported in Russia (76.9%) [26], in Morocco (68.6%) [45], in isolates of the LAM family in Cameroon [30] and also in Korea (49.1%) [46]. In INH^R^ strains, neither insertions nor complete deletions of *katG* were found, which is evidence of the rare occurrence of these mutations in clinical isolates, although they were reported previously by other authors [47,48]. Fourteen (31.8%) INH^R^ isolates did not show mutations at the four loci analyzed. This discrepancy between the phenotypic results and the genotypic drug resistance tests could be attributed to the presence of other mutations located either outside the selected target region or the selected genes. Several others studies have reported that mutations in *inhA* or its promoter region are usually associated with low-level resistance of INH. Moreover, INH-resistant isolates with *inhA* mutations can have additional mutations in *katG*, conferring higher levels of INH-resistance [11].

All mutations found in *fabG1*-*inhA* promoter region were not associated with phenotypic resistance. The substitution of G to C at position -47 first described by Homolka and al. [21] in an INH-resistant strain has been found in both susceptible (24/44; 54.5%) and resistant isolates (5/44; 11.4%). Thus, this mutation seems to not correlate with INH-resistance. The mutation -102 C → T not yet described is also not relevant to INH-resistance since it was found only in susceptible isolates.

The analysis of SM-resistance mechanism revealed that none of the SM-resistant strains carried a mutation in the *rrs* gene although those mutations have been described as main resistance mechanism that confer high level SM -resistance [12]. Clinical isolates showing no mutations in *rpsL* or *rrs* gene have been reported in the literature [49]. A previous investigation from Cameroon encountered *rpsL* or *rrs* mutations in SM-resistant isolates from the Central Region of Cameroon [50]. In contrast in the current investigation only *rpsL* mutations were associated with SM-resistance. This indicates that further studies are necessary to delineate the molecular markers for SM-resistance. Mutations in the *rpsL* locus have been hypothesized to be an alternative mechanism of SM-resistance like mutations in the *gidB*[51] or efflux pumps [13]. Overall, we detected *gidB* mutations in 18.5% of SM-resistant isolates and 6% of SM susceptible isolates. Although the encountered mutations in resistant samples were not observed in susceptible isolates, their association with SM-resistance needs to be confirmed.

Three contiguous genes encoding arabinosyl transferases and designated *embC, embA,* and *embB* were analyzed in the present study. These 3 genes have been identified in *M. tuberculosis*[52]. Previous studies based on limited sequencing region containing the *embCAB* genes have identified mutations that result in replacement of amino acid residues and are found only in EMB-resistant organisms cultured from humans [52]. In this study, the *embB* analysis gene identified 1 of 2 resistant isolates with EMB-resistance-associated nucleotide substitutions in codon 306ATG → GTG that result in amino acid replacement (Met → Val). This is in accordance with others studies analyzing EMB-resistant clinical isolates of *M. tuberculosis* that identified *embB* amino acid-conferring mutations in approximately 50 to 70% isolates with resistance-associated polymorphisms [52]. Certain variations affecting *embA* (330CTG → TTG) and *embC* (-20A → C and -230 A → C) appeared to be not associated with drug resistance. Given the low number of EMB-resistant isolates in our investigation further studies are needed to confirm these findings.

## Conclusion

This study provided the first molecular characterization of *M. tuberculosis* drug resistance in the Central Region of Cameroon using DNA sequencing. *rpoB* and *katG315* mutations known to be involved in resistance had high specificities and sensitivities for detecting RIF- and INH-resistance respectively. However, the correlation between molecular and phenotypic resistance testing for the determination of SM- and EMB-resistance was lower. This study clearly shows the need for continuous phenotypic and genotypic characterization of drug resistance at the national level in order to determine the most suitable molecular marker for drug resistance in our setting. The fact that mutations at codon *katG315* and at the *rpoB* gene show high specificities for resistance against INH or RIF respectively suggests that these may be suitable molecular marker for diagnostic test in Cameroon. Consequently the WHO recommended GeneXpert technology is appropriate for the detection RIF-resistance in the Central Region of Cameroon.

## Abbreviations

TB: Tuberculosis; MDR: Multi-drug resistance; SLD: Second-line drug; DOTS: Directly observed treatment short course; HIV: Human immunodeficiency virus; ATCC: American type culture collection; INH: Isoniazid; RIF: Rifampicin; SM: Streptomycin; EMB: Ethambutol; AST: Antimicrobial susceptibility testing; MIC: Minimal inhibitory concentration; DNA: Désoxyribonucleic acid; CANTAM: Central Africa Network on Tuberculosis, AIDS/HIV and Malaria; RRDR: Rifampicin resistant determining region.

## Competing interests

The authors declare there are no competing interests.

## Authors’ contributions

EMT and LKS contributed equally, they carried out all the molecular analysis as Ph.D students, participated in field work and drafted the manuscript. JPAA, JCT, ST, GGM, ALTW participated in field work and revised the manuscript. CK participated in the conception, design and supervision of field work. SE supervised mycobacteria culture and DST. FN is the coordinator and project manager of the CANTAM network. She revised the manuscript. VNPB is the Workpackage Leader of the CANTAM-TB project. She was the overall supervisor and chief designer of the project and critically revised the manuscript. MF is the Co-Workpackage Leader of CANTAM-TB project and Coordinator of the DAAD PAGEL-Program of the University of Tübingen. He designed and supervised the molecular analysis and critically revised the manuscript. All authors read and approved the final manuscript before submission.
